# Knowledge and Practice toward DEXA Scan among Women in Jazan City, Saudi Arabia

**DOI:** 10.3390/healthcare12151459

**Published:** 2024-07-23

**Authors:** Turki M. Dhayihi, Nawaf F. Haqawi, Sarah A. Hakami, Fahad M. Harthi, Sarah H. Moafa, Yahya M. Alawi, Yazeed B. Abutaleb, Nadim I. Areshy, Ali M. Hendi

**Affiliations:** Radiology Department, Faculty of Medicine, Jazan University, Jazan 45142, Saudi Arabia; nawafhaqawi@outlook.com (N.F.H.); sss0sss2001@gmail.com (S.A.H.); fahadharthi59@gmail.com (F.M.H.); sarhhhsn19@hotmail.com (S.H.M.); sirya021@gmail.com (Y.M.A.); abutalebyaz@gmail.com (Y.B.A.); moonar07@gmail.com (N.I.A.); dr.hendi@gmail.com (A.M.H.)

**Keywords:** osteoporosis, DEXA scan, menopause, women, Jazan, Saudi Arabia

## Abstract

***Background***: Osteoporosis, characterized by reduced bone mass and increased fracture risk, presents a significant public health challenge. Dual-energy X-ray Absorptiometry (DEXA) scans offer a reliable means of assessing bone mineral density (BMD) and detecting osteoporosis. ***The aim*** of this study is to assess awareness, attitude, and practice of women in the Jazan region toward DEXA scan. ***Methods***: Data were collected through a self-administered online questionnaire. Statistical analysis was conducted using SPSS version 29.0. The total knowledge scores of participants were calculated to assess potential associations with sociodemographic data. A *p*-value of less than 0.05 was considered statistically significant. ***Results***: Among 400 women, 230 (57.5%) had low knowledge scores primarily due to poor knowledge of the DEXA scan procedure. Merely 39 women had undergone a DEXA scan mainly due to medical prescription (*n* = 22, 56.4%). Total knowledge scores were significantly higher among women with high monthly incomes (*p* = 0.019) and those working in medical-related jobs (*p* = 0.017). ***Conclusions***: This study underscores the need for targeted interventions to improve awareness of DEXA scans among women in Jazan City. Additionally, the findings suggest that socioeconomic factors may influence awareness levels, emphasizing the importance of tailored educational strategies to reach diverse demographics.

## 1. Introduction

Osteoporosis is a prevalent condition characterized by a widespread decrease in bone mass, leading to weakened bones and an increased risk of fractures [1]. Dual-energy X-ray Absorptiometry (DEXA) is a method used to evaluate bone mineral density (BMD), and osteoporosis is diagnosed when the BMD T-score falls 2.5 or more standard deviations below the average of a young adult reference population. Approximately 40% of postmenopausal women of Caucasian descent experience osteoporosis, and given the aging demographic, this figure is projected to steadily rise in the coming years [2,3,4]. In Saudi Arabia, a few studies have estimated the prevalence of osteoporosis among adults ranging from 23.4% to 39.5%. However, most of these studies date back more than 15 years [5,6]. The most recent estimate of osteoporosis in Saudi Arabia is 52.8% of women and 63.6% of men in a hospital-based setting However, this was a screened population with a probable indication [7]. According to the International Osteoporosis Foundation, osteoporosis affects more than 200 million women worldwide, and one in three women over 50 will experience osteoporotic fractures [8]. The incidence of femur fractures caused by osteoporosis is approximately 4950/100,000 people-year for all individuals in Saudi Arabia [9]. Fractures resulting from osteoporosis significantly affect the quality of life, causing pain, limited mobility, and feelings of depression. Furthermore, mortality rates are elevated following hip or vertebral fractures associated with osteoporosis [10]. National Osteoporotic Foundation, USA and the national consensus group on osteoporosis for the Middle East and North Africa have identified several major risk factors for low BMD, including premature menopause; low physical activity; a family history of fractures; a personal history of fracture as an adult; cigarette smoking; alcohol consumption; thin build; use of oral glucocorticosteroid therapy for three months or longer; or a history of rheumatoid arthritis, thyroid disease, or liver disease [11,12,13]. The DEXA scan offers several advantages that make it a valuable tool in clinical practice. One of the primary advantages is its low radiation exposure to the patient, which is approximately 90% less than that of a standard chest radiograph [14]. This feature enhances the safety profile of the DEXA scan, making it suitable for repeated use in monitoring BMD over time [15]. Additionally, DEXA scan provides accurate results with a low scanning period, making it time-efficient and convenient for both patients and healthcare providers [16]. Overall, the advantages of the DEXA scan, including its safety, accuracy, efficiency, and utility in assessing BMD, underscore its significance in clinical settings. Numerous studies have been conducted that have assessed the knowledge of osteoporosis, particularly among women, and most of the sample population had a fair level of knowledge about osteoporosis [17]. In contrast, there has been insufficient research carried out in Jazan City that assesses awareness of the DEXA scan as a screening tool for osteoporosis, and with this research, we aimed to fill that void and direct future work on the same theme. The significance here lies in understanding the general public’s awareness and knowledge regarding the use of the DEXA scan as a screening tool for osteoporosis. By assessing public awareness, this study can identify gaps in knowledge and misconceptions, thereby informing targeted educational campaigns and interventions to improve understanding and promote the importance of the DEXA scan as a screening tool. Ultimately, enhancing public awareness can lead to early detection, timely treatment, and improved overall bone health outcomes in the population. So, the aim of this study is to assess the general public’s awareness and knowledge regarding the use of the DEXA scan as a screening tool for osteoporosis.

## 2. Materials and Methods

### 2.1. Study Design: Cross-Sectional Study

Study setting: This study was carried out in the Jazan region, including its 14 governorates, which is located on the Red Sea coast in Saudi Arabia’s southwest corner, with the number of women in Jazan City at about 601,500. 

### 2.2. Study Population

- Inclusion criteria: All women aged 18 years and above were informed about the requirements of this study, and those living in Jazan communities and use Arabic language were selected.
- Exclusion criteria: Individuals who refused to participate or failed to complete the questionnaires, women less than age of 18, non-Jazan women, or those who did not use Arabic language were excluded from this study.

### 2.3. Method and Data Collection

Sampling method: Convenience sampling in a nonrandom fashion.

Sample size calculation: Estimating that the number of women in Jazan City is 601,500, the sample size was thus 420 using http://www.raosoft.com/samplesize.html (accessed on 20 February 2024) with a 95% confidence interval, 5% margin of error, and a 10% nonresponse rate. The underlying assumption for this calculation is that the characteristic being assessed has a prevalence of 50%.

Data were collected through an online validated questionnaire sent to the participants by the authors. The questionnaire was designed to assess the level of public awareness and practices among women in Jazan City concerning bone health and DEXA scans. It included 18 questions about knowledge of DEXA scans and bone health, as well as 3 questions about practices regarding DEXA scans. Participants were recruited through various channels, including social media, community groups, and local organizations, ensuring a wide reach within the Jazan region.

### 2.4. Ethical Consideration

All potential participants were informed about the objectives of this study at the beginning of the questionnaire. They were assured that no harm is expected to occur if they decide to participate in this study. Data were anonymously collected and kept secure. Their consent to participate was requested in a writing form at a mandatory section at the beginning of the questionnaire. Ethical approval from Jazan University Research Ethic Committee (HAPO-10-Z-001) was taken.

### 2.5. Statistical Analysis Plan

This study aims to assess the level of public awareness and practices among women in Jazan City concerning bone health and DEXA scans. Additionally, it aims to identify the factors that influence their knowledge about DEXA scans. 

The analysis comprised both descriptive and inferential statistical methods. Since all variables are categorical, frequencies and percentages were utilized to describe their distribution. The survey included 18 questions to examine women’s knowledge about DEXA scans (8 questions) and bone health (10 true or false questions). For the knowledge score, represented as a continuous variable, medians and interquartile ranges are provided. Additionally, participants were asked about their practices regarding DEXA scans through three questions. To assess significant differences in participants’ knowledge scores based on sociodemographic features, the Mann–Whitney test (for two independent groups) and the Kruskal–Wallis test (for more than three groups) were utilized. Nonparametric tests were chosen due to the nonnormal distribution of the numeric variables, as confirmed by the Shapiro–Wilk test (*p* < 0.05). Statistical significance was determined at a *p*-value less than 0.05, with a 95% confidence interval. Data entry and analysis were performed using IBM SPSS version 29.

## 3. Results

Our study aims to evaluate women’s knowledge and practices concerning DEXA scans in Jazan City, Saudi Arabia. A total of 400 women consented to participate in this study. The majority fell within the 18–25 age bracket (*n* = 184, 46.0%), although women of other age groups were also found. A significant proportion held a university degree (*n* = 263, 65.8%); however, a considerable number were unemployed (*n* = 251, 62.7%), with 47.4% of participants reporting a monthly household income of 5000 Saudi Riyal or below (*n* = 177), while the remainder earned more than 5000 Saudi Riyal. The marital status was almost evenly distributed between single (*n* = 189, 47.3%) and married (*n* = 181, 45.3%) participants, with the remaining being divorced or widowed. Among the married participants (*n* = 211), over half reported having been pregnant more than three times (*n* = 117, 55.5%), and a majority relied on alternative feeding methods for their babies aside from breastfeeding (*n* = 139, 65.8%) (Table 1).

The survey comprised 18 questions assessing participants’ knowledge. Those who correctly answered 10 questions or more were considered as highly knowledgeable, and vice versa. Overall, the knowledge scores of the 400 participants were below average, with a mean score of 8.96 and a median score of 9. Upon further classification, only 170 participants (42.5%) demonstrated a high level of knowledge by answering 10 or more questions about DEXA scans, while the majority 230 (57.5%) exhibited low knowledge of the topic by correctly answering 9 or fewer questions (Table 2).

Women’s knowledge about bone health was evaluated, and their responses are summarized in Table 3. Most women recognized that osteoporosis affects both men and women (*n* = 375, 93.8%); it can be exacerbated by high caffeine and low calcium intake (*n* = 370, 92.5%). Additionally, many participants understood that premature menopause typically leads to increased bone loss (*n* = 330, 82.5%) and recognized the importance of calcium intake for women not taking estrogen (*n* = 328, 82%). Furthermore, a substantial proportion acknowledged the availability of treatment options for osteoporosis (*n* = 282, 70.5%) and the positive impact of activities like walking (*n* = 240, 60%) and high-impact exercise (*n* = 225, 56.3%) on bone health, as the decline in bone mass starts after the age of 30 (*n* = 240, 60%). However, fewer participants were aware that lower-weight women are more susceptible to osteoporosis than heavier women (*n* = 160, 40%), and that hormonal replacement therapy after menopause can mitigate bone loss (*n* = 144, 36%).

Women were also assessed for their knowledge about DEXA scans, as presented in Table 4. Overall, a poor level of knowledge was observed regarding this topic. Only 43% (*n* = 172) of participants had heard of DEXA scans. Merely 24.3% (*n* = 97) were aware that DEXA scans are primarily used to assess bone density. Additionally, only 5% (*n* = 20) knew that DEXA scans are recommended for individuals aged 65 years or older. Furthermore, very few participants were aware that medical conditions such as postmenopausal status, family history of osteoporosis, history of fractures, and glucocorticoid use necessitate a DEXA scan for assessing bone mass density (*n* = 131, 32.8%). Only a minority knew that patients should remove any jewelry or metallic objects before the start of the procedure (*n* = 100, 25%), and that the procedure requires the patient to lie on the table while the scanner passes over the body (*n* = 76, 19%). The highest level of knowledge was about the scan’s contraindications such as pregnancy, recent nuclear medicine, and presence of any metallic implants (*n* = 242, 60.6%).

Regarding women’s practices toward DEXA scans, only 9.8% (*n* = 39) had undergone the procedure, while 90.2% (*n* = 361) had not. Among those who underwent the procedure, 22 (56.4%) reported that it was recommended by a doctor, while only 2 (5.2%) were advised by a family member or a friend. Three women (7.7%) had a history of fractures and wanted to know their BMD, and the remaining 12 (30.7%) had other reasons for undergoing the scan. Among those who did not undergo the DEXA scan, the reasons included lack of awareness about the procedure (*n* = 162, 44.8%), it was not recommended by a doctor (*n* = 167, 46.3%), inaccessibility of the service (*n* = 23, 6.3%), fear (*n* = 5, 1.4%), or the high cost of the procedure (*n* = 4, 1.2%) (Table 5).

The results of the analysis revealed significant associations between participants’ knowledge scores and some sociodemographic features (Table 6). Specifically, employment status (*p* = 0.017) and monthly household income (*p* = 0.019) showed significant associations with knowledge scores. Women employed in medical-related jobs demonstrated higher knowledge scores regarding DEXA scans and bone health, with a median score of 10 and an interquartile range (IQR) of 7.50–12, while unemployed women had the lowest knowledge scores with a median of 9.00 and an IQR of 7.00–10.00. Similarly, participants with higher monthly household incomes (10,000–15,000 SAR) exhibited greater knowledge scores, with a median score of 10 and an IQR of 8–11, whereas women with a low income (<5000 SAR) had the lowest knowledge score of 8.00 with an IQR of 7.00–10.00. Conversely, factors such as age, education level, marital status, the number of pregnancies, and the number of breastfed infants did not show significant associations with knowledge scores regarding DEXA scans (*p* > 0.05).

The potential associations between DEXA scan performance and participants’ sociodemographic data were explored. Only age showed a significant association with DEXA scan performance (*p* = 0.043), with older women in the age groups of 46–55 and 56–65 being more likely to have undergone a DEXA scan (16.7% and 22.2%, respectively) compared with other age groups. Other sociodemographic data did not show any significant associations with DEXA scan performance among participants (Table 7).

## 4. Discussion

Osteoporosis is a major public health concern especially with the increase in elderly population; its prevalence increases with age, as, for women, it increases from 2% at 50 years to 25% at 80 years [18]. Osteoporosis prevalence in the Kingdom of Saudi Arabia (KSA) was estimated to be 63.3% in men and 58.4% in women; these numbers demonstrate a huge burden of osteoporosis on the healthcare system [7]. Normally, by the third decade of life, bone resorption starts to exceed bone formation, which results in a net loss of bone mass leading to an increased bone fragility and risk of fracture [19]. Osteoporosis is a silent disease that goes undiagnosed until complications occur, mainly osteoporotic fragility fractures caused by minor trauma as a result of the BMD; the fragility fractures mainly affect the spine, forearm, hip, and shoulder [18].

Diagnosis of osteoporosis was made by a BMD radiological study using DEXA assessment [20]. However, given the nature of the disease and its late presentation, screening is crucial to decrease the incidence of complications. According to the U.S. Preventive Services Task Force (USPSTF), all women aged 65 years and older should be screened, preferably using DEXA of the hip and lumbar spine [21]. Thus, in this community-based cross-sectional study, we assessed the awareness of the women of Jazan City toward the DEXA scan and the reasons determining their practices.

According to our results, the majority of responders had good knowledge about bone health, with most women knowing that osteoporosis affects both men and women (*n* = 375, 93.8%), and that high caffeine and low calcium intake increase the risk of osteoporosis (*n* = 370, 92.5%). On the other hand, the lowest level of knowledge was about postmenopausal hormonal therapy with most women not realizing its role in slowing down bone loss (*n* = 256, 64.0%). This is consistent with the results of A. Alamri et al. who studied the knowledge, attitude, and practice of Saudi population toward osteoporosis, with most participants showing a fair level of knowledge with a significantly higher level of knowledge among women compared with men [22]. Our findings are also consistent with what was previously reported by Al-Rawi et al., which was a cross-sectional study assessing the knowledge and awareness among the Saudi residents regarding the DEXA scan and osteoporosis [8]. They reported that most of the participants have a good level of knowledge regarding bone health and the DEXA scan. Although that study included men and women, there was a significant difference in the level of knowledge between the two sexes, with women showing a higher level of knowledge [8]. They also stated that most participants have not heard about the DEXA scan, which is similar to our findings. However, in contrast to Al-Rawi et al., we assessed the knowledge about the DEXA scan separately, and we found that most women had generally a low level of knowledge about the DEXA scan, with the highest level of knowledge being about the scan’s contraindications such as pregnancy, recent nuclear medicine, and presence of any metallic implants (*n* = 239, 60.6%), while the lowest level of knowledge being about the recommended age for the DEXA scan, which is 65 years old or above (*n* = 20, 5.0%). These findings emphasize the urgent need for awareness campaigns for women to increase their knowledge about the importance of osteoporosis screening in general and the role of hormonal therapy for postmenopausal women to lower the incidence of osteoporosis complications and reduce its burden on the healthcare system.

Upon assessing women’s practice toward the DEXA scan in Jazan, we found that most women had not undergone a DEXA scan before (*n* = 361, 90.2%). However, most of the participants were young, but we found that the primary reasons were that it was not recommended by a doctor (*n* = 167, 46.3%) or due to a lack of awareness about the procedure (*n* = 162, 44.8%). These findings are consistent with those of Al-Rawi et al., which demonstrated that the majority of participants had not undergone a DEXA scan, and among those who did, it was often based on a recommendation from a doctor [8]. This suggests that medical advice plays a crucial role in determining whether individuals are aware of the procedure. Furthermore, it is important to assess the physician’s attitude and practice toward osteoporosis and the DEXA scan and how often do they recommend DEXA scanning. Ahmed et al. investigated regarding osteoporosis among general practitioners in Al Majmaah, KSA, and reported that most respondents exhibited sufficient knowledge of the disease. Moreover, most of them were familiar with the role of plain X-rays for fracture prediction, while only a few had accesses to perform BMD tests [23]. Similarly, Yehia et al. conducted a study in which the majority of physicians recognized plain X-rays as useful for fracture prediction. Additionally, most were aware that DEXA scans are the optimal diagnostic method for osteoporosis and can help predict fracture risk and determine BMD. Interestingly, less than one-third of physicians acknowledged that their patients were adequately informed about osteoporosis [24].

The results of our analysis revealed a significant association between participants’ knowledge scores and employment status (*p*-value = 0.017), showing higher knowledge scores among women in medical-related jobs. This is similar to the results of Al-Rawi et al. that showed a relatively higher level of knowledge among employees and students in the medical field [8]. Moreover, we noticed significantly higher knowledge scores among women attaining high monthly incomes (*p*-value = 0.019), particularly participants with a monthly income of 10,000 to 15,000 SAR, while those getting less than 5000 SAR/ month scored lower scores. Contrarily, another study reported that participants within the middle-income category had significantly higher levels of awareness about osteoporosis compared with those in the lower and high-income categories [8].

The relationship between age and participants’ level of knowledge regarding osteoporosis appears to be subject to controversy. While Hurst et al. underscored that older age is linked with heightened knowledge [25], A. Alamri et al. showcased a higher level of knowledge among younger individuals [22]. Despite this study not finding a significant association between age and levels of knowledge about osteoporosis and DEXA scans, it did show a significant association between age and the performance of DEXA scans (*p* = 0.043). Older women underwent DEXA scans more than younger ones, which might be attributed to their higher awareness level compared with younger women as revealed in Hurst et al. [25].

The association between individuals’ level of education and their knowledge about osteoporosis appears to yield conflicting findings across different studies. For instance, Raizah et al. reported that highly educated individuals had poor knowledge about osteoporosis [26], whereas Al-Rawi et al. found a higher level of knowledge among those with higher education [8]. In our study, we did not observe a significant association between knowledge scores and participants’ level of education despite the majority of our participants holding a bachelor’s degree. This discrepancy in findings underscores the complexity of factors influencing individuals’ understanding of osteoporosis and highlights the need for further research to elucidate these relationships.

### Limitations of the Study

While our study provides valuable insights into the knowledge and practices of women in Jazan City regarding osteoporosis and the DEXA scan, it is important to acknowledge several limitations. Firstly, the use of an online questionnaire may have led to a sample biased toward younger, more educated individuals, potentially limiting the generalizability of our findings to the broader population. Additionally, reliance on self-reported data introduces the possibility of response biases and inaccuracies. Moreover, the cross-sectional design of this study prevents the establishment of causal relationships between variables. Furthermore, the convenience sampling method utilized may not fully represent the diversity of the population in the Jazan region. Lastly, as all participants were women, we lack insight into the perspectives of men on this topic. Despite these limitations, this study sheds light on an important public health issue and suggests avenues for further research and intervention.

## 5. Conclusions

This study showed that the overall knowledge level of women was relatively low due to their poor understanding of the DEXA scan procedure. However, further investigations revealed that the knowledge level was significantly higher among women employed in medical-related jobs and those with high monthly household incomes when compared with their counterparts.

## Figures and Tables

**Table 1:** Sociodemographic Data (*n* = 400).

Variable:		*n* (%)
Age (years)	18–25	184 (46.0)
	26–35	60 (15.0)
	36–45	84 (21.0)
	46–55	54 (13.5)
	56–65	18 (4.5)
Education	Primary	14 (3.5)
	Intermediate	13 (3.3)
	Secondary	92 (23.0)
	Bachelor’s	263 (65.8)
	Postgraduate	18 (4.5)
Marital status	Single	189 (47.3)
	Married	181 (45.3)
	Divorced	17 (4.3)
	Widow	13 (3.3)
Number of pregnancies (*n* = 211)	No	25 (11.8)
	1	30 (14.2)
	2	39 (18.5)
	≥3	117 (55.5)
Number of breast-fed children (*n* = 211)	0	139 (65.8)
	1	26 (12.3)
	2	9 (4.5)
	≥3	37 (17.4)
Employment status	Unemployed	251 (62.7)
	Self-employed	12 (3.0)
	Working in a medical field	41 (10.3)
	Working in a nonmedical field	96 (24.0)
Monthly income (SAR)	≤5000	177 (47.2)
	5000–10,000	73 (19.5)
	10,000–15,000	62 (16.5)
	≥15,000	27 (7.2)

*n*: frequency, %: percentage.

**Table 2:** Knowledge Level of Participants (*n* = 400).

	Mean (SD)	Median (IQR)
Sum Knowledge Score	8.96 (2.45)	9.00 (7–11)
Knowledge Level	High	Low
*n* (%)	170 (42.5)	230 (57.5)

*n*: frequency, %: percentage, IQR: interquartile range.

**Table 3:** Knowledge Regarding Bone Health (*n* = 400).

Question:	Correct Answer	*n* (%)
Osteoporosis affects both men and women	True	375 (93.8)
2. High caffeine and low calcium intake increase risk of osteoporosis	True	370 (92.5)
3. Normally bone loss speed-up after menopause	True	330 (82.5)
4. After menopause, women not on estrogen need about 1500 mg of calcium (5 glasses of milk) daily	True	328 (82.0)
5. There are treatments for osteoporosis after it develops	True	282 (70.5)
6. Most people gain bone mass after the age of 30	False	240 (60.0)
7. Walking has a great effect on bone health	True	240 (60.0)
8. High impact exercise improves bone health	True	225 (56.3)
9. Lower-weight women have osteoporosis more than heavier women	True	160 (40.0)
10. Replacing hormones after menopause cannot slow down bone loss	False	144 (36.0)

*n*: frequency, %: percentage.

**Table 4:** Knowledge Regarding DEXA Scan (*n* = 400).

Question:	Correct Answer	*n* (%)
11. Have you heard about DEXA scan before?	Yes	172 (43.0)
12. What is the primary purpose of DEXA scan?	To assess bone density	97 (24.3)
13. What is the recommended age for DEXA scan?	65 and above	20 (5.0)
14. What medical conditions required DEXA scan screening?	- Postmenopausal women - Family history of osteoporosis - History of fractures - Glucocorticoid use	131 (32.8)
15. What precautions that person should take before undergoing DEXA scan?	Removing metal objects or jewelry	100 (25.0)
16. What does a DEXA scan procedure involve?	Lying on the table while the scanner passes over the body	76 (19.0)
17. How frequently should individuals with normal bone mineral density undergo DEXA scans for routine screening?	Every 3–5 years	53 (13.3)
18. What conditions prevent someone from undergoing DEXA scan?	- Pregnancy - Recent nuclear medicine - Metal implants	242 (60.6)

*n*: frequency, %: percentage.

**Table 5:** Practices Toward DEXA Scan (*n* = 400).

Question:		*n* (%)
Did you preform DEXA scan before?	Yes	39 (9.8)
	No	361 (90.2)
2. If yes, why? (*n* = 39)	Doctor prescribed it	22 (56.4)
	Friend or family member suggested for me	2 (5.2)
	Had history of fracture and wanted to know bone mineral density	3 (7.7)
	Other causes	12 (30.7)
3. If no, why? (*n* = 361)	Lack of awareness	162 (44.8)
	Cost of procedure	4 (1.2)
	Wasn’t recommended by doctor	167 (46.3)
	Accessibility issues	23 (6.3)
	Fear	5 (1.4)

*n*: frequency, %: percentage.

**Table 6:** Relationship Between Sociodemographic Data and Knowledge Scores of Participants.

Variable:		Median (IQR)	*p*-Value *
Age (years)	18–25	9.00 (7.00–11)	0.564
	26–35	9.00 (7.00–10.75)	
	36–45	9.00 (7.00–11)	
	46–55	9.50 (8.00–11.00)	
	56–65	9.50 (6.00–10.00)	
Education	Primary	8.00 (4.75–9.50)	0.111
	Intermediate	8.00 (6.50–10.00)	
	Secondary	9.00 (7.00–10.00)	
	Bachelor’s	9.00 (7.00–11.00)	
	Postgraduate	10.50 (7.00–12.00)	
Marital status	Single	9.00 (7.00–11.00)	0.784
	Married	9.00 (7.00–11.00)	
	Divorced	9.00 (7.50–10.50)	
	Widow	9.00 (7.00–10.00)	
Number of pregnancies	No	9.00 (7.00–11.00)	0.351
	1	9.00 (7.00–11.00)	
	2	9.00 (7.00–11.00)	
	≥3	9.00 (7.00–11.00)	
Number of breast-fed children	0	9.00 (7.00–11.00)	0.228
	1	8.00 (6.75–11.25)	
	2	9.00 (7.50–12.00)	
	≥3	8.00 (6.50–10.50)	
Employment status	Unemployed	9.00 (7.00–10.00)	0.017
	Self-employed	9.00 (7.25–10.00)	
	Working in a medical field	10.00 (7.50–12.00)	
	Working in a non-medical field	10.00 (7.25–11.0)	
Monthly income (SAR)	≤5000	8.00 (7.00–10.00)	0.019
	5000–10,000	9.00 (7.00–10.00)	
	10,000–15,000	10.00 (8.00–11.00)	
	≥15,000	9.00 (7.00–11.00)	

*: Kruskal Wallis test, IQR: Inter-Quartile Range.

**Table 7:** Associations Between Sociodemographic Data and Practices Toward DEXA scan.

Variable:		Did You Perform DEXA Scan before?		*p*-Value
		Yes	No	
Age (years)	18–25	13 (7.1)	171 (92.9)	0.043 ^f^
	26–35	3 (5.0)	57 (95.0)	
	36–45	10 (11.9)	74 (88.1)	
	46–55	9 (16.7)	45 (83.3)	
	56–65	4 (22.2)	14 (77.8)	
Education	Primary	1 (7.1)	13 (92.9)	0.167 ^f^
	Intermediate	1 (7.7)	12 (92.3)	
	Secondary	14 (15.2)	78 (84.8)	
	Bachelor’s	20 (7.6)	243 (92.4)	
	Postgraduate	3 (16.7)	15 (83.3)	
Marital status	Single	16 (8.5)	173 (91.5)	0.635 ^f^
	Married	19 (10.5)	162 (89.5)	
	Divorced	2 (11.8)	15 (88.2)	
	Widow	2 9 (5.4)	11 (84.6)	
Employment status	Unemployed	19 (7.6)	232 (92.4)	0.084 ^f^
	Self-employed	1 (8.3)	11 (91.7)	
	Working in a medical field	3 (7.3)	38 (92.7)	
	Working in a non-medical field	16 (16.7)	80 (83.3)	
Monthly income (SAR)	≤5000	4 (5.3)	71 (94.7)	0.216 ^c^
	5000–10,000	12 (9.2)	118 (90.8)	
	10,000–15,000	14 (14.7)	81 (85.3)	
	≥15,000	9 (9.0)	91 (91.0)	

f: fisher’s exact test, c: chi-square test.

## Data Availability

All data will be made available by the authors upon request.
